# Supplementary material for: Tailored Synthesis of Catalytically Active Cerium Oxide for N, N-Dimethylformamide Oxidation
Source: Materials (Basel). 2023 Jan 16;16(2):865. doi: 10.3390/ma16020865 (PMC9867243; doi:10.3390/ma16020865)
Supplement: Supplementary file 1 [file materials-16-00865-s001.zip › materials-2021096-supplementary.pdf]

Supplementary Material

Tailored Synthesis of Catalytically Active Cerium Oxide for N, N-Dimethylformamide Oxidation

Cedric Karel Fonzeu Monguen [1,2,†], En-Jie Ding [1,3,†], Samuel Daniel [1,2], Jing-Yang Jia [1,3], Xiao-Hong Gui [3] and Zhen-Yu Tian [1,2,*]

[1] Institute of Engineering Thermophysics, Chinese Academy of Sciences, Beijing 100190, China; karel@iet.cn (C.K.F.M.); dingenjie@iet.cn (E.-J.D.); samuel@iet.cn (S.D.); jiajingyang@iet.cn (J.-Y.J.)
[2] University of Chinese Academy of Sciences, Beijing 100049, China
[3] School of Emergency Management and Safety Engineering, China University of Mining and Technology, Beijing 100083, China; gxhbox@sina.com
* Correspondence: tianzhenyu@iet.cn; Tel./Fax: +86-10-8254-3305
† These authors contributed equally to this work.

Table of Contents

Section S1: Synthesis method of as-prepared samples 2

Section S2: Experimental setup for catalytic tests 3

Section S3: Catalyst structure 4

Section S4: Catalytic performance 5

References 6

Section S1: Synthesis method of as-prepared samples

Cerium oxides (CeOx) was prepared using sol-gel method. The different reaction occurs during the preparation are:

- Step 1: Dissolution of the precursor for homogeneity
- Step 2: Hydrolysis of the precursor in NaOH
- Step 3: condensation using ethanol and ammoniac sodium
- Step 4: Vaporization of the solvent and the gel formation
- Step 5: calcination and growth of the particle

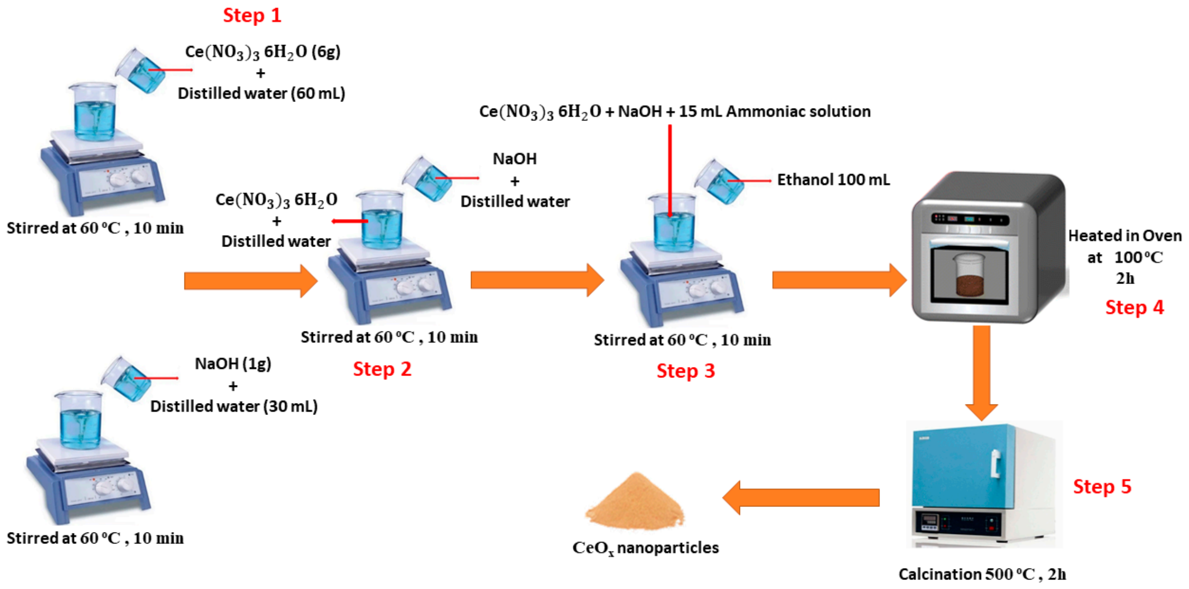

Figure S1. Sol-gel setup for the nanoparticles catalyst preparation [1, 2].

Section S2: Experimental setup for catalytic tests

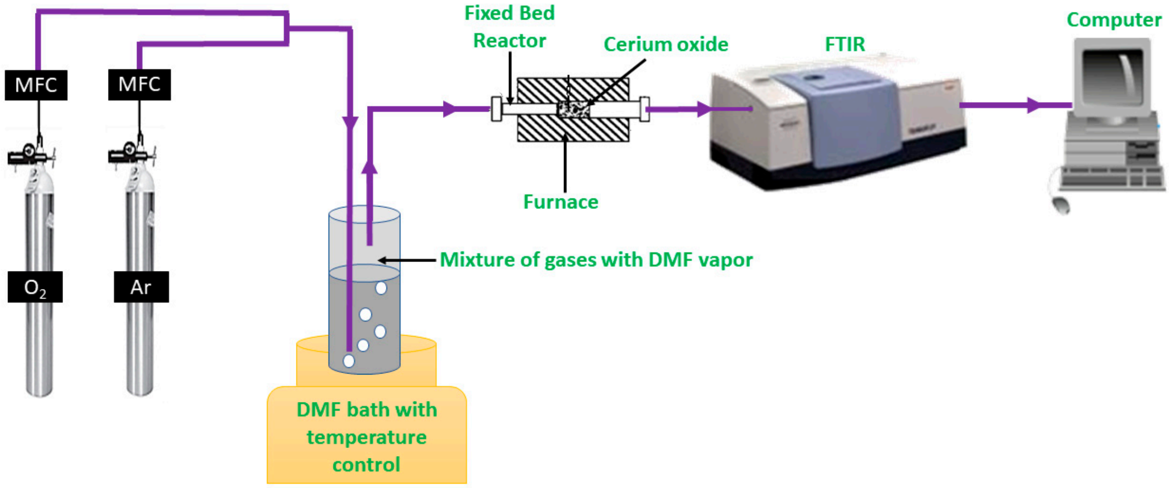

Figure S2. The catalytic test system is connected to the FTIR setup for the exhaust gas analysis.

Section S3: Catalyst structure

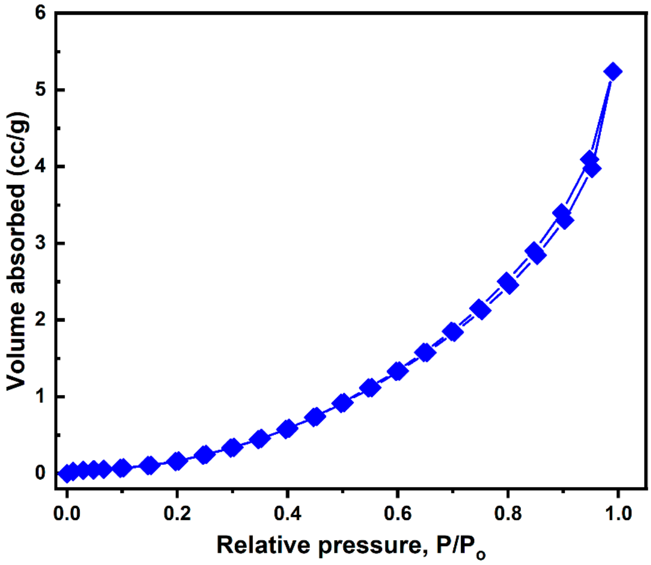

Figure S3: BET surface area analysis of CeO_x: Adsorption-desorption isotherms curves.

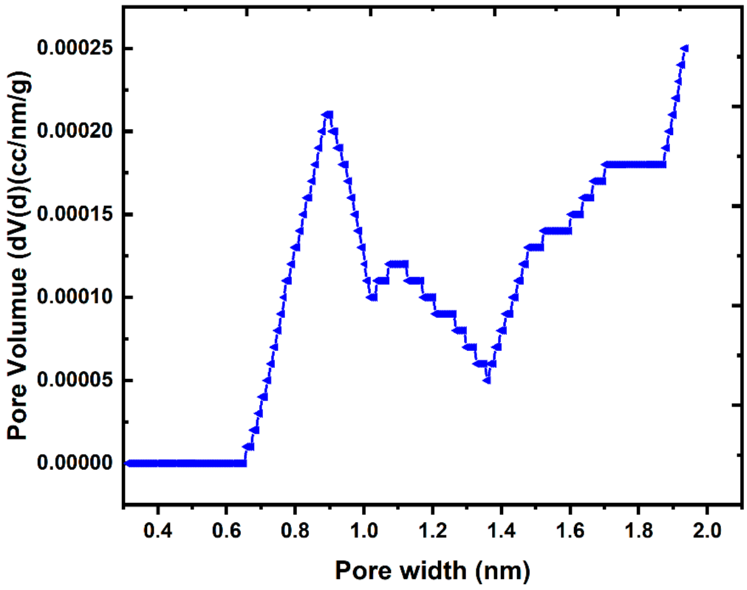

Figure S4: Pore size distribution.

Section S4: Catalytic performance

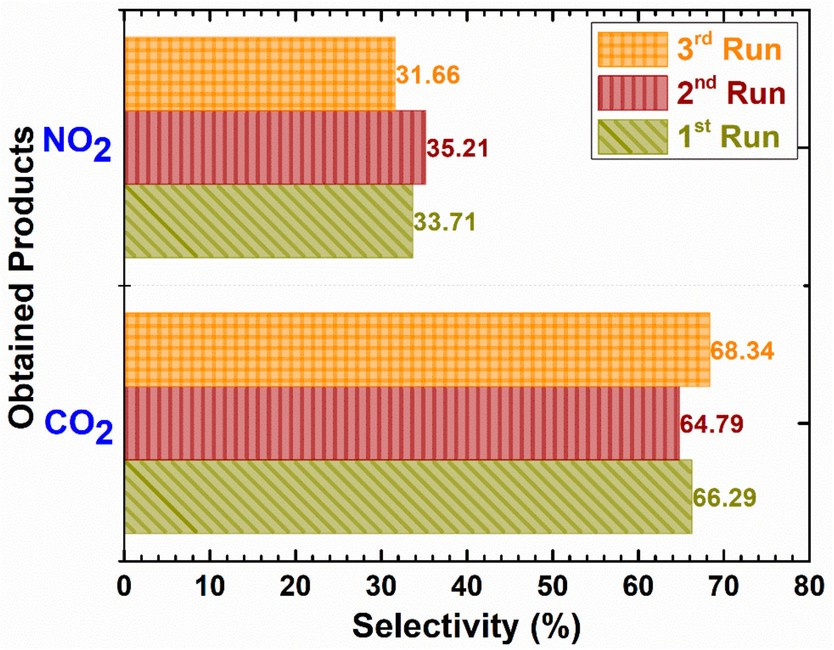

Figure S5: Reproducibility of the selectivity of the products.

References

[1] Fonzeu Monguen, C.K.; El Kasmi, A.; Arshad, M.F.; Kouotou, P.M.; Daniel, S.; Tian, Z.Y. Oxidative Dehydrogenation of Propane into Propene over Chromium Oxides. *Ind. Eng. Chem. Res.* **2022**, *61*, 4546–4560. https://doi.org/10.1021/acs.iecr.2c00813.

[2] Daniel, S.; Monguen, C.K.F.; El Kasmi, A.; Arshad, M.F.; Tian, Z.Y. 2022. Oxidative Dehydrogenation of Propane to Olefins Promoted by Zr Modified ZSM-5. *Catal. Lett.* **2022**, 1–15. https://doi.org/10.1007/s10562-022-03977-6.
